# Case report: Complete and durable response to larotrectinib (TRK inhibitor) in an infant diagnosed with angiosarcoma harbouring a *KHDRBS1-NTRK3* fusion gene

**DOI:** 10.3389/fonc.2023.999810

**Published:** 2023-02-23

**Authors:** Catherine Cervi, Zoltán Sápi, Gábor Bedics, Erik Zajta, Lajos Hegyi, Judit Pápay, Katalin Dezső, Edit Varga, Katalin Mudra, Csaba Bödör, Monika Csóka

**Affiliations:** ^1^ Department of Pathology and Experimental Cancer Research, Semmelweis University, Budapest, Hungary; ^2^ 2^nd^ Department of Paediatrics, Semmelweis University, Budapest, Hungary; ^3^ HCEMM-SE Molecular Oncohematology Research Group, Department of Pathology and Experimental Cancer Research, Semmelweis University, Budapest, Hungary

**Keywords:** precision oncology, next-generation sequencing (NGS), comprehensive genomic profiling (CGP), paediatric soft tissue sarcoma, angiosarcoma, neurotrophic tyrosine receptor kinase (NTRK), *NTRK* fusion gene, larotrectinib

## Abstract

Significant improvements in the survival rates of paediatric cancer have been achieved over the past decade owing to recent advances in therapeutic and diagnostic strategies. However, disease progression and relapse remain a major challenge for the clinical management of paediatric angiosarcoma. Comprehensive genomic profiling of these rare tumours using high-throughput sequencing technologies may improve patient stratification and identify actionable biomarkers for therapeutic intervention. Here, we describe the clinical, histopathological, immunohistochemical and molecular profile of a novel and precision medicine-informed case where a *KHDRBS1-NTRK3* fusion determined by next-generation sequencing-based comprehensive genomic profiling led to complete and sustained remission (clinical and radiological response) in an otherwise incurable disease. Our patient represents the first paediatric angiosarcoma harbouring a targetable *NTRK3* fusion in the literature and demonstrates the first example of targeting this alteration in angiosarcoma using larotrectinib, an *NTRK* inhibitor. Clinical and radiological remission was achieved in under two months of therapy, and the patient is currently in complete remission, 4 month after stopping larotrectinib therapy, which was given over 17 months with only mild side effects reported. Therefore, this remarkable case exemplifies the true essence of precision-based care by incorporating conventional pathology with the why, when, and how to test for rare oncogenic drivers and agnostic biomarkers in paediatric angiosarcoma.

## Introduction

Soft tissue sarcomas are a heterogeneous group of malignant tumours derived from mesenchymal cells, accounting for approximately 8-10% of all paediatric malignancies, 0.3% of which are angiosarcomas (1–3). Paediatric angiosarcomas are highly aggressive malignant neoplasms associated with a poor prognosis due to their lack of response to conventional therapy. Due to the heterogeneous genetic profile of primary angiosarcoma, collaborative studies such as the Angiosarcoma Project play an integral role in categorising and applying novel and targeted therapies in this tumour entity (4). However, this project focuses on adult angiosarcoma cases, and little is known about molecular profiling and the application of targeted therapy in paediatric angiosarcoma. The tropomyosin receptor kinase (Trk) family consists of TrkA, TrKB and TrKC transmembrane receptors which are encoded by *NTRK1*, *NTRK2* and *NTRK3* genes, respectively (5). *NTRK* gene fusions are agnostic biomarkers associated with several adult and paediatric solid tumours. In 2018, the FDA approved small-molecule inhibitors of the TRK kinases due to their efficacy regardless of tumour entity, patient age and performance status (6–9). Moreover, due to lowered cost and routine application of next-generation sequencing (NGS) testing in the clinical setting, our knowledge of tumours that harbour *NTRK* fusions, targetable with TRK inhibitors are expanding, improving our understanding of rare tumours and enabling precision-based therapeutic management of clinically challenging tumours (10–12). Here, we describe the first case of paediatric angiosarcoma harbouring a targetable *NTRK3* fusion in the literature and demonstrate the first example of targeting this alteration in paediatric angiosarcoma.

## Case report

### Clinical presentation

An eight-month-old girl presented to her family practitioner with life-threatening respiratory symptoms. One week later, she was admitted to ICU with hypoxemia, and tachydyspnoea, caused by bilateral pleural effusion and consequent dystelectasis of the lung area. At that time, bilateral chest tubes were performed, and 200 ml of fluid was drained from both sides. A significant amount of pleural effusion appeared every 2-3 days, which required drainage several times. MRI (22/01/21) confirmed the presence of an infiltrating lesion surrounding the trachea, pharynx, superior mediastinum, pleura, chest wall and bilateral pleural effusion. Histological, cytological and immunohistochemical examinations were performed on a neck soft tissue biopsy and pleural fluid aspiration samples, confirming the diagnosis of paediatric angiosarcoma, morphological grade 2. At the time point of diagnosis, therapeutic strategies were limited. From a clinical standpoint, the patient presented in a critical condition and no curative option was available since local therapy, on account of the disease’s extent and the child’s age. Based on the conventional pathological diagnosis and due to the aggressive behaviour of the tumour, the Cooperative Weichteilsarkom Studiengruppe (CWS)-2012 chemotherapy protocol was applied between 04/02/21 and 08/04/21; considering the patient’s age, paclitaxel was initially excluded from the regimen. She received 2 courses of vincristine-adriamycin-cyclophosphamide (VAC). However, due to her clinical condition and the increasing amount of fluid, 4 cycles of paclitaxel were promptly initiated based on the decision of the tumour board. Only a moderate clinical and radiological response was observed to the paclitaxel therapy, with the patient permanently requiring drainage and intensive care.

### Cytological and histopathological examinations

**Figure 1:**
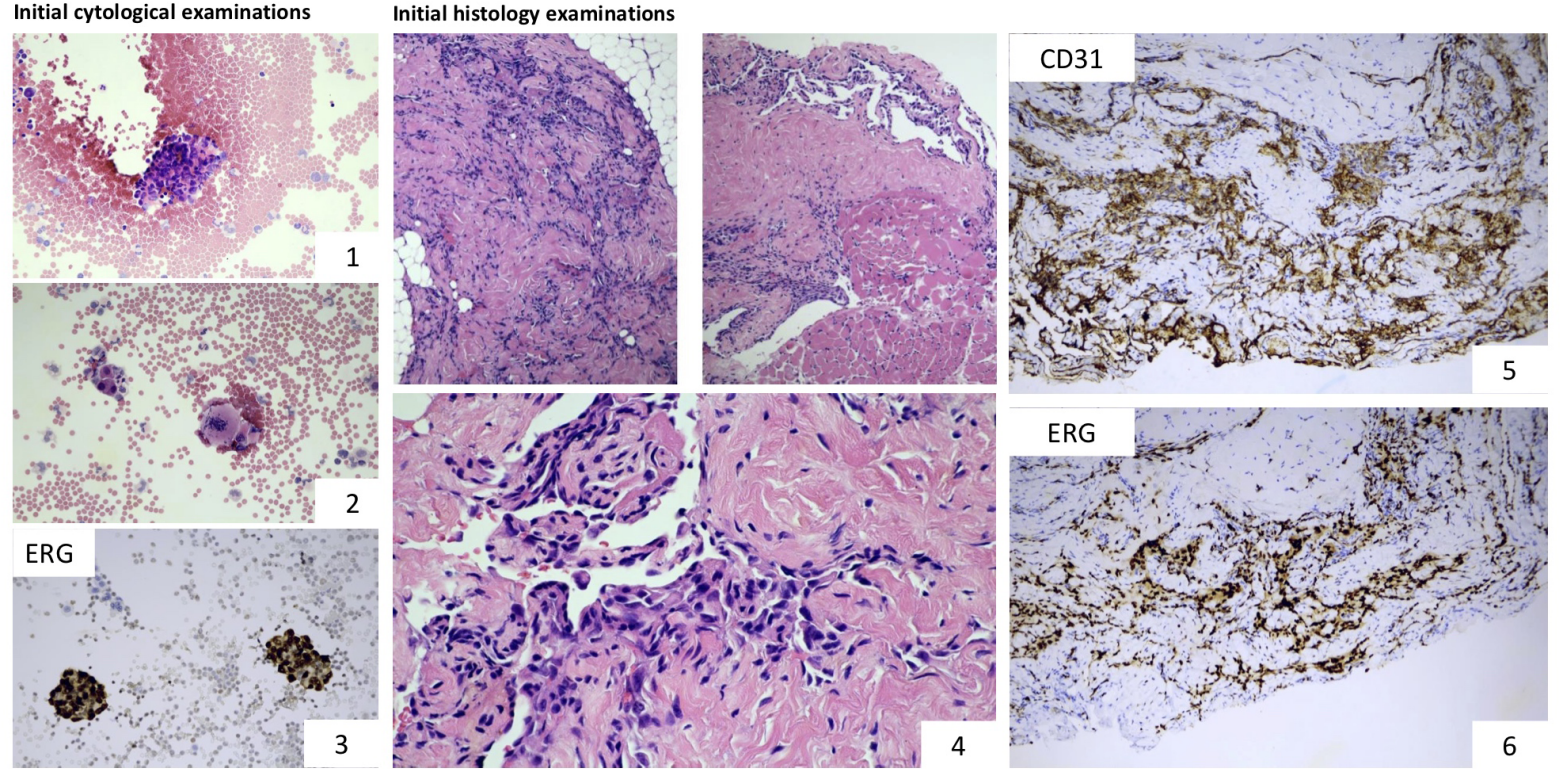
Microscopic examinations: Cytological and immunocytochemical examinations performed on the aspirated pleural fluid show the presence of tumour cells (images 1 and 2) and ERG nuclear positivity (image 3). Characteristic histological features of angiosarcoma: proliferating atypical tumour cells forming anastomosing vascular channels. The lining endothelial cells display marked atypia (image 4). Tumour cells showed strong cytoplasmic CD31 and nuclear ERG positivity (images 5 and 6). Based on the morphology and the immunophenotype, the lesion corresponded with the diagnosis of angiosarcoma, grade 2.

**Pleural effusion specimen**: dissociated atypical tumour cells sometimes arranged in small clusters were observed. Immunocytochemistry showed Vimentin and ERG positivity while WT1 and CAMTA1 were negative. INI1 (SMARCB1) was retained. All these were suggestive of angiosarcoma (**Figure 1**). **Histology** (biopsy from neck/pleural region): anastomosing vascular spaces lined by atypical flattened endothelial cells with endothelial multilayering were observed infiltrating fat and striated muscle. Immunohistochemistry showed strong ERG nuclear positivity and CD31 cytoplasmic positivity (**Figure 1**). Cytokeratin (AE1-AE3) and CD34 were negative and no WT1 and CAMTA1 nuclear positivity was observed. Ki-67 proliferation index was 20%. INI1 (SMARCB1) was retained ruling out the possibility of rhabdoid tumour (one of the most common paediatric soft tissue malignancies of this age). The lack of characteristic intracytoplasmic vacuoles and CAMTA1 nuclear immunopositivity ruled out epithelioid haemangioendothelioma. The diagnosis of angiosarcoma (grade 2) was established.

### Comprehensive genomic profiling

**Figure 2:**
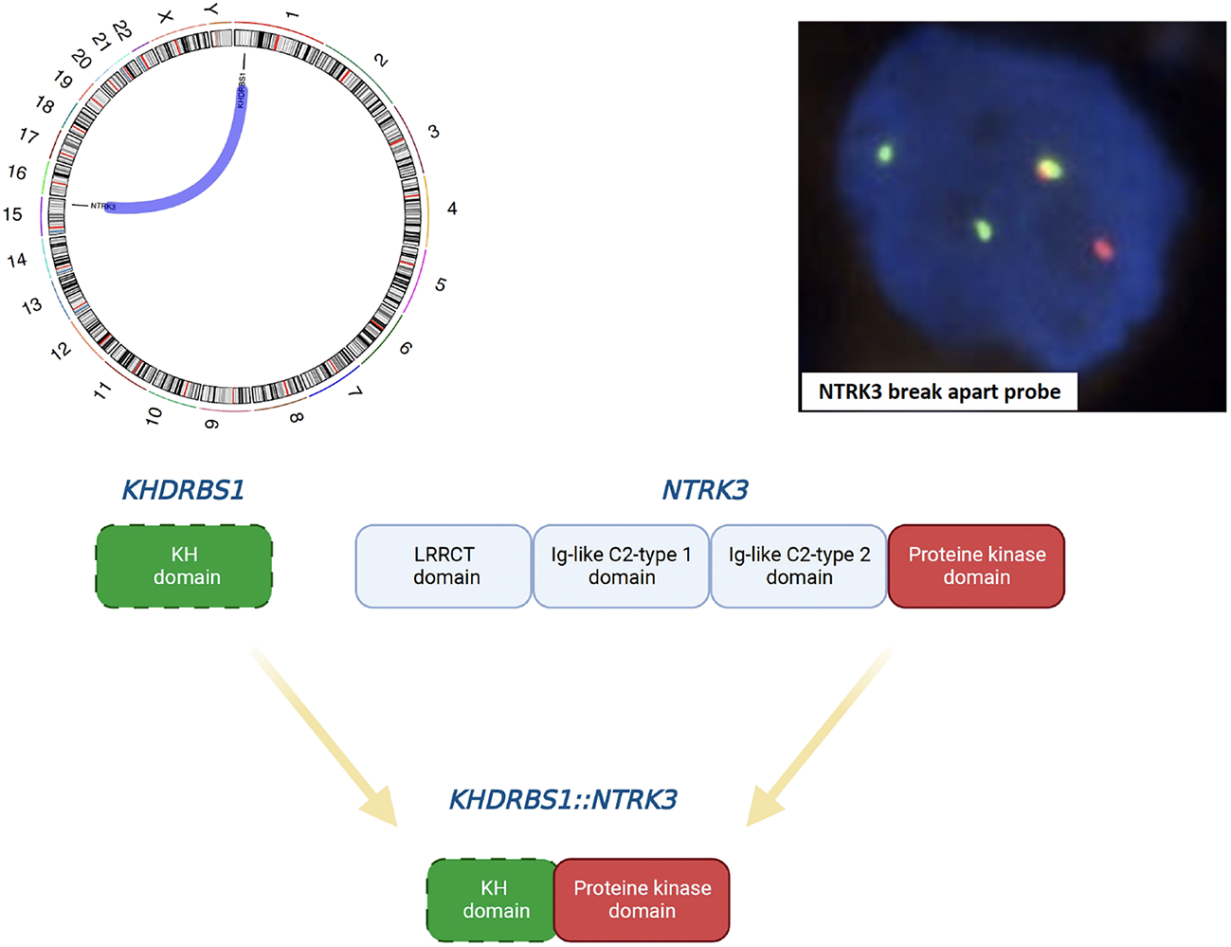
Schematic illustration of the in-frame *KHDRBS1-NTRK3* fusion (Circos plot and schematic diagram were created using MAFTOOLS and Biorender, respectively) and confirmation using the *NTRK3* break apart FISH probe. The following breakpoints were identified using the FusionCatcher software: chr1:32039573A and chr15:88033045 for *KHDRBS1* and *NTRK3*, respectively.

Comprehensive genomic profiling from DNA and RNA samples extracted from the formalin-fixed paraffin-embedded specimen was performed using the Illumina TruSight Oncology 500 assay on a NextSeq2000 (Illumina) next-generation sequencing platform, in accordance with the manufacturer’s instructions. Small insertion/deletions (InDel) or single nucleotide variants (SNV) were detected in the *BRCA1* (c.3700_3704delGTAAA, p.V1234fs*8) and *RNF43* (c.1976delG, p.G659fs*41) genes, respectively. Low-level (<5-fold) amplifications were detected in the *ALK, RET, FGF3, FGF6, EGFR, FGF4, BRCA2*, and *MYCL* genes. An in frame *KHDRBS1-NTRK3* gene fusion, previously unreported in angiosarcoma, was detected, offering therapeutic intervention with a TRK inhibitor (**Figure 2**). This fusion was validated by fluorescence *in situ* hybridisation (**Figure 2**).

### Molecularly targeted therapy and clinical follow-up

**Figure 3:**
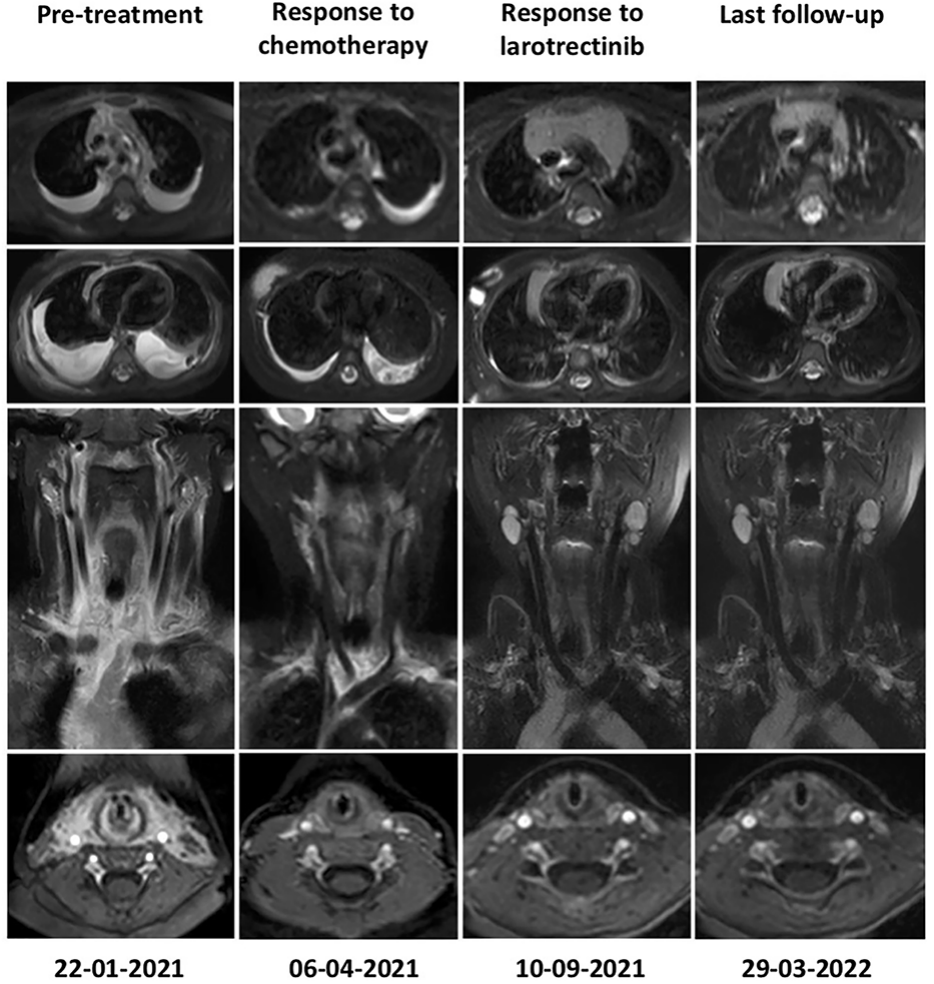
Radiological response as determined by Magnetic Resonance imaging before treatment (22.01.21), following administration of the CWS 2012 protocol (06.04.21) and NTRK inhibitor therapy (Larotrectinib) (10.09.21), respectively. Follow-up MRI is performed every three months; the latest images show a sustained response to Larotrectinib therapy as no visible tumour mass is detectable (29.03.22). While a limited therapeutic response upon completion of chemotherapy, a remarkable and sustained complete remission from Larotrectinib was observed.

Based on the *KHDRBS1-NTRK3* fusion, on-label use of larotrectinib (VITRAKVI) (per os 100 mg/m2 twice daily, commencing on 21/04/21) was initiatied. The patient was diligently monitored using clinical, neurological, and radiological assessments. The clinical response to larotrectinib therapy was evident during routine follow-up examinations. At the time of presentation, the patient scored a low Karnofsky performance status score of 20, requiring frequent drainage of her extensive bilateral pleural effusion. Notably, the patient’s Karnofsky score significantly improved throughout her treatment trajectory; Karnofsky scores of 40 (following chemotherapy) and 90 (within one week of starting larotrectinib), where she was discharged home without the need for further drainage. To date, the patient has undergone 17 months of larotrectinib therapy, and her current performance status is 100 (mild side effects, normal development, average height, and weight gain were reported). The only noted adverse side effects to larotrectinib were observed in May 2022; grade 2 liver toxicity. Following the CTCAE version 4.03 protocol, the drug dosage was reduced, and the liver toxicity was resolved. On the 12^th^ of July 2022, the patient received the full dosage again. The patient completed her Larotrectinib treatment on the 19^th^ of September 2022 and is still in complete remission (January 2023). Overall, a remarkable and sustainable clinical and radiological response to larotrectinib has been achieved. (**Figure 3**).

## Discussion

Paediatric angiosarcoma is exceedingly rare and aggressive in behaviour compared to cases diagnosed in adulthood. The CWS-2012 international protocol is clear regarding the treatment (chemotherapy, local surgery and or radiotherapy), diagnostic steps, the staging classification, and the follow-up regimen (13). In this case, local therapy was excluded due to the tumour’s extent and location; only a moderate response to chemotherapy was achieved using the CWS-2012 protocol. Thus, genomic profiling was performed to identify actionable variants and expand therapeutic possibilities. In light of the paucity of genomic profiling or the application of targeted therapy in paediatric angiosarcoma, we present an unprecedented challenging case and demonstrate a durable clinical response to TRK inhibitor (larotrectinib) based upon a precision oncology approach using next-generation sequencing-based comprehensive genomic profiling.

In normal physiology, neurotrophins are TRK ligands, where ligand binding plays an integral role in several key functions; proliferation and survival of neuronal cells, maintenance of the central and peripheral nervous system, and regulation of behaviour, sensation, movement, and cognition (14–16). *NTRK* gene fusions are the most common mechanism of TRK oncogenic activation found in both adult and paediatric cancers. As a rule of thumb, the frequency of *NTRK* fusions in paediatric oncology is common among rare tumours and rare among common tumours. However, despite the availability of TRK inhibitor therapy owing to FDA approval in 2018, no data has been published on the application of TRK inhibitor therapy in the management of paediatric angiosarcoma. Additionally, the clinical success of TRK inhibitors, the long-term side effects and the permanent response to these inhibitors, particularly in infants and children, are limited to case reports (17). Fortunately, secondary resistance has only been reported in a minority of patients treated with larotrectinib (6, 18). Clinical trials in large cohorts demonstrate the marked and durable anti-tumour activity of larotrectinib in adult and paediatric tumours harbouring *NTRK* fusions (locally advanced or metastatic solid tumours regardless of patient age, performance status or tumour entity). Recent evidence suggests that larotrectinib is well-tolerated in adults and children, with predominantly grade 1-2 adverse effects (19). A growing body of studies demonstrates the prevalence and spectrum of *NTRK* fusion partner genes in paediatric soft tissue sarcoma. A previous case report detailed the finding of a *KHDRBS1-NTRK3* rearrangement found in a congenital CD34+ skin tumour. However, TRK inhibition therapy was not provided based on this finding (20). Overall, it is noticeable that different frequencies are shown across these various studies, most likely due to differences in available diagnoses and referral bias (21, 22). Considering these studies, a paradigm shift towards genetic profiling of rare tumours is paramount to identify clinically targetable biomarkers, improve patient stratification and expand therapeutic options in these clinically challenging tumours.

This case study demonstrates the first application of TRK inhibitors in managing paediatric angiosarcoma. Since there are no guidelines regarding the duration of therapy or follow-up regimens of paediatric patients undergoing TRK inhibitor therapy, further large-scale trials are required. Ongoing specific digital-droplet PCR assays coupled with close clinical and radiological assessments will enable continuous monitoring of the measurable residual disease from serial liquid biopsy specimens to assess the depth of the achieved remission and detect relapse or resistance mutations.

## Conclusion

Paediatric angiosarcoma is a rare subtype of soft tissue sarcoma associated with a poor prognosis and limited therapeutic options. To our knowledge, we present the first case of precision-based management of paediatric angiosarcoma harbouring a *KHDRBS1-NTRK3* fusion gene that responded to an *NTRK* inhibition. The routine application of next-generation sequencing of rare paediatric malignancies associated with poor prognosis expands the morphological spectrum and clinical relevance of targeted therapies, especially in cases that do not respond to conventional therapy. Furthermore, the true essence of precision-based care by incorporating conventional pathology with the why, when, and how to test for oncogenic drivers and agnostic biomarkers in rare soft tissue sarcomas is demonstrated throughout this unprecedented case.

## Data availability statement

The original contributions presented in the study are included in the article/supplementary materials, further inquiries can be directed to the corresponding author/s.

## Ethics statement

The studies involving human participants were reviewed and approved by Regional, Institutional Scientific and Research Ethics Committee, Semmelweis University. Written informed consent was obtained from the individual(s) and minor(s)’ legal guardian/next of kin to publish any potentially identifiable images or data in this article.

## Author contributions

CC, CB and MC wrote the manuscript. EZ, CC, LH and GB performed sequencing experiments and analyzed data. ZS, JP, KD, EV, KM and MC contributed with pathological analysis and participated in the clinical management of the patient. All authors contributed to the article and approved the submitted version.
